# Metallic Effects on p-Hydroxyphenyl Porphyrin Thin-Film-Based Planar Optical Waveguide Gas Sensor: Experimental and Computational Studies

**DOI:** 10.3390/nano12060944

**Published:** 2022-03-13

**Authors:** Nuerguli Kari, Marco Zannotti, Rita Giovannetti, David Řeha, Babak Minofar, Shawket Abliz, Abliz Yimit

**Affiliations:** 1 Institute of Applied Chemistry, College of Chemistry, Xinjiang University, Urumqi 830046, China; nurri7695@163.com; 2 Chip Research Center, Chemistry Division, School of Science and Technology, University of Camerino, 62032 Camerino, Italy; 3 Laboratory of Structural Biology and Bioinformatics, Institute of Microbiology of the Czech Academy of Sciences, Zamek 136, 37333 Nove Hrady, Czech Republic; reha@nh.cas.cz; 4 Key Laboratory of Coal Cleaning Conversion and Chemical Engineering Process, College of Chemistry and Chemical Engineering, Xinjiang University, Urumqi 830046, China; shawket_abliz@sina.com

**Keywords:** planar optical waveguide, nitrogen dioxide gas sensor, porphyrin complex, molecular dynamic simulations, quantum mechanical calculation

## Abstract

Metal effects on the gas sensing behavior of metal complexes of 5,10,15,20-tetrakis(4-hydroxyphenyl)porphyrin (THPP) thin film was investigated in terms of detecting NO_2_ gas by the planar optical waveguide. For this purpose, several THPP and metal complexes were synthesized with different central metal ions: Co(II), Ni(II), Cu(II), and Zn(II). Planar optical gas sensors were fabricated with the metalloporphyrins deposited on K^+^ ion-exchanged soda-lime glass substrate with the spin coating method serving as host matrices for gas interaction. All of the THPP complex’s films were fully characterized by UV-Vis, IR and XPS spectroscopy, and the laser light source wavelength was selected at 520 and 670 nm. The results of the planar optical waveguide sensor show that the Zn–THPP complex exhibits the strongest response with the lowest detectable gas concentration of NO_2_ gas for both 520 nm and 670 nm. The Ni–THPP and Co–THPP complexes display good efficiency in the detection of NO_2_, while, on the other hand, Cu–THPP shows a very low interaction with NO_2_ gas, with only 50 ppm and 200 ppm detectable gas concentration for 520 nm and 670 nm, respectively. In addition, molecular dynamic simulations and quantum mechanical calculations were performed, proving to be coherent with the experimental results.

## 1. Introduction

As one of the nonlinear optical limiters, porphyrin is a potential choice to fabricate a guiding wave-light system. Based on the electron-rich properties and macromolecular structure, this type of molecule attracts research interest and is applied in detecting gases and volatile organic compounds. Central metal effects on the nonlinear optical behavior and the excited state dynamics of tetrahydroxyphenylporphyrins (THPPs) with Fe, Co, Ni, Zn metals were investigated utilizing open aperture Z-scan technique and pump-probe experiments [1].

Odorants interact with tissues in living creatures, and metalloproteins are regarded as the sensing part of the olfactory system [2]. Therefore, colorimetric sensor arrays were developed as an optoelectronic nose to “see the smells”—differentiating volatile organic compounds in a facile way with an ordinary scanner based on metal substituted porphyrins [3]. Metalloporphyrins were also used as one of the responsive materials in the colorimetric sensor array to identify combustion residues according to the color change [4], which is also used to identify explosives due to the ligand ability of metalloporphyrins [5]. Moreover, volatiles emitted from fungi and metabolism changes after the addition of fungicides was also observed with this array [6]. This type of sensor was studied to assess the freshness of meat such as beef, shrimp, fish, chicken, and pork [7] and to identify coffee aroma [8]. Selected responsive dyes deposited on a hydrophobic membrane in a colorimetric sensor array were used to analyze beers [9], drinks such as Pepsi [10], and to detect sweeteners at millimolar concentrations [11]. Furthermore, Zn-substituted, bis-pocketed porphyrins, based on ortho-substitution of the tetraphenylporphyrin (TPP), were used to discriminate volatiles according to their size and shape [12].

Planar optical waveguide sensor (OWGS) is a facile method to detect molecules due to the interaction between sensing materials and evanescent fields formed with laser lights [13,14]. Different research studies focus on the fabrication of different types of sensors applying pH indicators [15], polyacrylate resin thin film [16], cyclodextrin polymer films [17], metal oxides, and [18] porphyrin dyes [19]. The film–analyte interaction mechanism and output light intensity has been restricted to the macroscopic view. Recently, our studies have focused on the effect of substituents from a microscopic view using molecular simulation [20]. In this work, the effect of metal centers in p-hydroxyphenyl substituted porphyrin thin film, with various transition metals, in optical waveguide towards NO_2_ gas, was studied with instrumental ways, molecular dynamics simulation, and quantum mechanical calculation.

## 2. Materials and Methods

### 2.1. Fabrication of THPP Metalloporphyrin Thin Film

5,10,15,20-tetrakis(4-hydroxyphenyl)porphyrin (THPP) was purchased from Sigma-Aldrich (Shanghai, China), and the metalloporphyrins, M–THPP (M = Co, Ni, Cu, and Zn), was synthesized using the procedure [21]. Thin films of the Co-, Ni-, Cu-, and Zn–THPP porphyrin complexes were fabricated, depositing each solution onto a side of K^+^ ion-exchanged soda-lime glass to improve waveguide forming ability. The ion-exchanged glass substrate with K^+^ ion-exchanged layer depth, approximately 1–2 μm, was prepared using soda-lime glass (76 × 26 × 1 mm) stored in melted KNO_3_ at 400 °C for 40 min to exaggerate the refractive index of the upper layer and help to form guiding light under the sensing film in optical waveguide detection system.

Spin coater was set (500 rpm, 5 s) as the first rotating speed and (1300 rpm, 25 s) as the second, simultaneously providing a vacuum condition. Thin-film width was 1 mm for all the metalloporphyrins. The porphyrin films and solutions were characterized by UV-Vis spectroscopy, and each film was characterized by UV-1780 ultraviolet spectrophotometer (SHIMADZU, Kyoto, Japan) and FT-IR spectroscopy (Alpha II, Bruker Co., Karlsruhe, Germany)

### 2.2. Characterization of Metalloporphyrin Film-Gas Interaction

**Figure 1:**
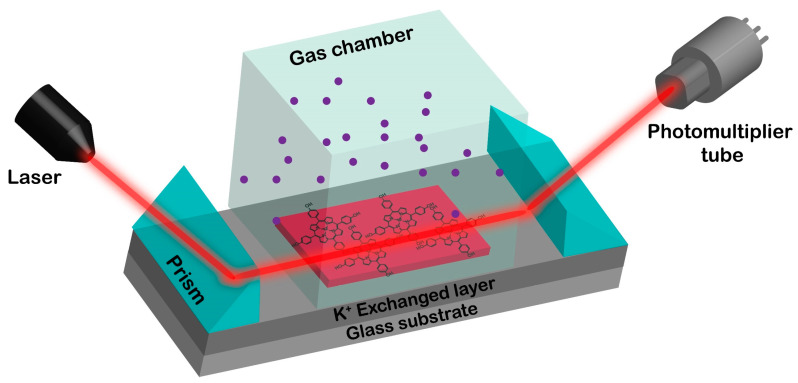
Self-assembled optical waveguide detection system based on THPP porphyrin film.

Optical waveguide detecting experiment was operated on a self-assembled detection system (Figure 1) by using the metal-complexes porphyrin thin film as sensor. In order to reduce the optical loss, a coupling diiodomethane material (n = 1.74) was used between the prisms (n = 1.79) and the substrate waveguide (n = 1.52). For the interaction between the gases and the film, a gas chamber with 2 cm^3^ of volume was used, and the injection of the gas was performed with a plastic syringe. The concentration of the gas inside the chamber was adjusted by the addition of dry air. In order to restore the initial state of the metalloporphyrins, air atmosphere was used after the interaction with the gases, for restoring Zn-THPP Trimethylamine (TMA) was used.

Semiconductor laser tubes (520 nm and 670 nm, 10 mW, Shenzhen Gainlaser Laser Technology Co., Ltd., Shenzhen, China) and a gas detection tube (working range: 2–200 ppm, Gastec, Beijing Municipal Institute, Beijing, China) were used.

### 2.3. Molecular Dynamic Simulations and Quantum Mechanical Calculations

Classical molecular dynamics (MD) simulations and quantum mechanical calculations (QM) have been used to understand the interaction of NO_2_ gas with THPP porphyrin with different metal ions at the molecular level.

MD simulations were performed in slab geometry in a box with the size of 5 × 5 × 20 (nm)^3^ with one porphyrin molecule and 2000 molecules of NO_2_. By performing MD simulations, we would like to investigate the affinity of porphyrin molecule toward different NO_2_ gas when the porphyrin molecule have complexed with different metal ions, namely Zn^2+^, Co^2+^, Ni^2+,^ and Cu^2+^. From MD simulations, possible orientation and conformations could be captured during the simulations, which can be used for higher accuracy quantum mechanical calculations. As different metal ions are used to form complexes with porphyrin, the complexes may have different affinities due to the different electronic structures of metal ions in interaction with NO_2_ gas.

For performing MD simulations, the General Amber Force Field (GAFF) model [22] shows good properties for inorganic and organic gases and liquid molecules [23,24,25,26,27].

Ab initio geometry optimization using the Gaussian 09 package [28] employing the B3LYP/cc-pVDZ method flowed by Restrained Electrostatic Potential (RESP) [29] using the Antechamber program [30] was used to calculate the partial charges of porphyrin and NO_2_ molecules. Each system with different metal ions was prepared by putting single porphyrin and 2000 NO_2_ molecules into the simulation randomly by application of Packmol program [31,32].

Steepest descent minimization procedure was used for minimization of all systems. After minimization, 500 ps NVT ensemble (Canonical ensemble) and NPT (isothermal–isobaric ensemble), restrained simulations were used to equilibrate the systems. Systems obtained after NPT simulation were used to build slab geometries. Linear constraint solver (LINCS) algorithm [33] was employed for all bonds involving hydrogen atoms. The short-range non-bonded interactions were truncated with the cutoff distance of 1.2 nm, and the long-range part of the electrostatic interactions was treated by the particle mesh Ewald method [34]. All the molecules in all systems, according to Maxwell–Boltzmann distribution at 300 K, were used to produce initial results [35] with the coupling constant of 0.1 ps to ensure constant temperature and pressure. Simulations were run for the production phase in NVT ensemble for 50 ns at 300 K with a time step of 2 fs. All simulations were performed employing Gromacs 4.6.5 program package (University of Groningen, Groningen, Netherlands) [36,37,38]. A Visual molecular dynamics (VMD) program was used for visualizations of the trajectory and preparation of snapshots [39].

For further investigation of the NO_2_ interaction with porphyrin molecules, which are complexed with different metal ions, namely Zn^2+^, Co^2+^, Ni^2+^, and Cu^2+^, quantum mechanical calculations have been performed.

For Zn metal ion complex of porphyrin with NO_2_ molecules, two different conformations were extracted from MD simulations were optimized by the density functional theory (DFT) method with B3LYP functional using 6-31G * basis set in Gaussian09 program(Gaussian, Inc., Carnegie Mellon University, Pittsburgh, PA, USA) [28]. The two conformations were capturing situations where nitrogen of NO_2_ was interacting with Zn^2+^ (conformation 1) or oxygen from NO_2_ was interacting with Zn^2+^ (conformation 2). The resulting structures were used as models for the preparation of other complexes. For other metal ion complexes, Cu^2+^, Co^2+^, and Ni^2+^ ions substituted the Zn^2+^ metal ion. Additionally, the structure with no metal ion present in the structure of porphyrin was prepared for the comparison of the interactions where the system is negatively charged. The geometries of the systems were optimized by the DFT method with B3LYP functional using 6–31G * basis set. The spin multiplicity of the systems was set to take into account the NO_2_ radical as well as spin multiplicity of metal-porphyrin complexes [40]. The interaction energies were calculated by the DFT method with B3LYP functional using 6–31G * basis set. Basis set superposition error (BSSE) was corrected using the counterpoise method of Boys and Bernardi [41].

## 3. Results and Discussion

### 3.1. Characterization of THPP Metalloporphyrin Complex

**Figure 2:**
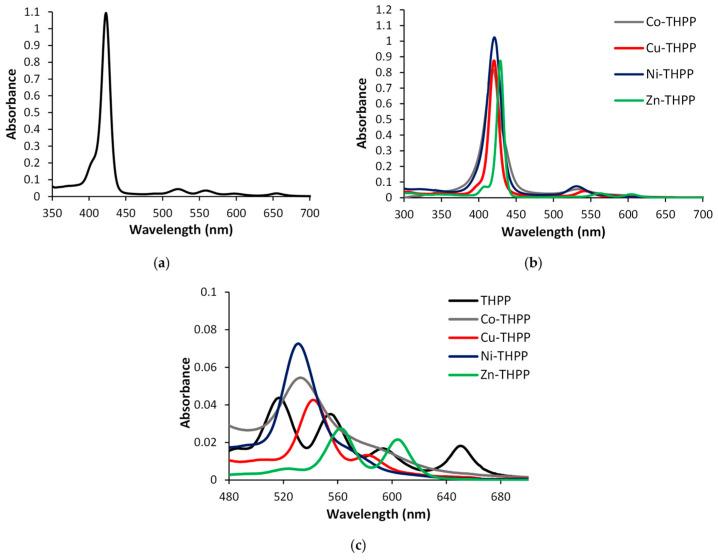
UV-Vis absorption spectra of (**a**) THPP-free porphyrin, (**b**) M–THPP (M = Co, Ni, Cu, Zn) complexes in DMF solution, (**c**) Q- bands magnification of THPP and metal complexes in DMF.

The UV-Vis absorption spectra of free-base THPP and the relative metal complexes with Co, Ni, Cu, and Zn in DMF are shown in Figure 2. All the complexes exhibit the B band (Soret band) at around 400 nm and two Q bands (β and α bands) in the range of 500–700 nm; the obtained spectra indicate the successful complexation of THPP.

In the free-base porphyrin, the Q bands are due to the transitions from the ground state to the two vibrational states of the excited state Q (0,0) and Q (1,0), and the presence of two hydrogen atoms in the free-porphyrin ring split the Q bands into four bands due to the vibrational modes: Qx (0,0), Qy (0,0), Qx (1,0) and Qy (1,0) (Figure 2a,c). In metalloporphyrins, the presence of a metal in the porphyrin ring leads to a change in geometry from D_2_h to D_4_h and degeneration of the x and y components, with the final result of only two bands in the Q region (Figure 2b,c) [42,43].

The molar extinction coefficient (ε) of each THPP–metal complex was calculated and reported in Table 1.

Among the four metal–THPP complexes, Zn–THPP presents the highest molar extinction coefficient (5 × 10^8^ M^−1^ cm^−1^), which is thousands of times higher than other metalloporphyrins (10^5^ M^−1^ cm^−1^), indicating that it has outstanding light absorption ability and potential application in optical gas sensor fabrication. At the same time, in the DMF solution, the wavelength of the Soret band of Zn–THPP is longer than that of other metalloporphyrins, as shown in Figure 2, showing that the HOMO-LUMO energy level varies to a different extent. Moreover, the observation of the Q region shows that the two Q bands, typical of the metal complexes of porphyrins, show differences with Zn–THPP, where the Q-band at lower energy is more pronounced with respect to the other THPP complexes. These results suggest a different molecular configuration of the zinc complex with respect to copper, cobalt, and nickel metalloporphyrins. The relative intensities of the Q bands can therefore correlate with the stability of the metal complex.

**Figure 3:**
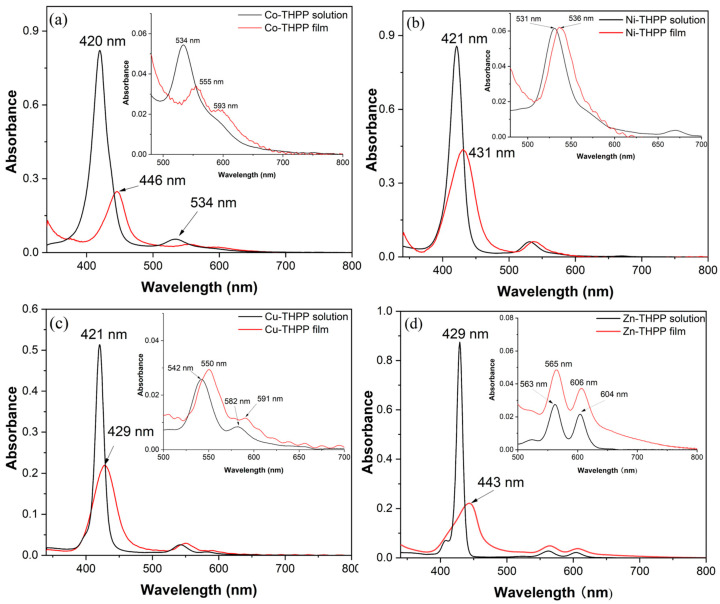
UV-Vis absorption spectrum of (**a**) Co–THPP, (**b**) Ni–THPP, (**c**) Cu–THPP and (**d**) Zn–THPP complex in solution (DMF) and in film state.

As shown in Figure 3, when metal–THPP complexes were deposited as films on the surface of K^+^ ion-exchanged glass substrate, Soret bands are red-shifted and widened for different degrees compared with each solution state. All the spectroscopic data for the metal–THPP film related to Soret bands are resumed in Table 2. Meanwhile, Q bands shifted to a longer wavelength synchronously. The observed spectral change of the Soret Band of all metalloporphyrins is due to the formation of J-aggregates on the glass surface [43]. Without changing their shape, the shifts in the Q-bands towards a higher wavelength demonstrate that the metalation of the porphyrin ring remains stable after the deposition on surfaces of glass.

**Figure 4:**
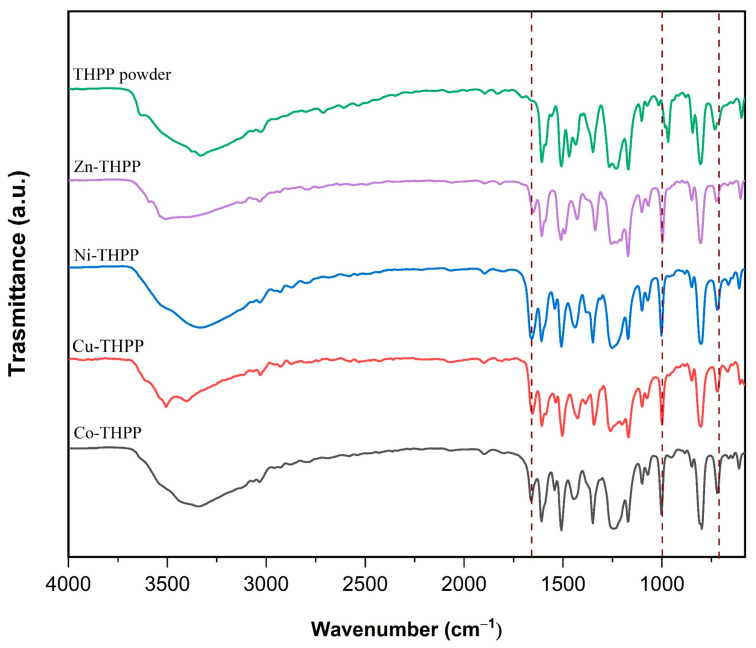
FT-IR spectrum of Co, Ni, Cu, Zn porphyrin complexes and THPP powder.

The infrared spectrum of each metalloporphyrin is shown in Figure 4. It was found the presence of the bands at around 3300 cm^−1^ relative to -NH, -CH phenyl, and -CH pyrrole stretching and of two bands at 1600 and 1350 cm^−1^ due to the vibration of the benzene ring. At 1510 cm^−1^ and, always, at 1350 cm^−1,^ the bands are assigned to the vibrations of C = N and C−N bonds, respectively [44]. The bands relative to the in-plane δ N−H and out of plane N−H for THPP porphyrin are at 973 and 710 cm^−1^. When a metal ion is inserted into the porphyrin ring, these bands disappear, and the characteristic functional groups of the metal-N bond are observed from the formation of new bands at around 1000 cm^−1^; this band corresponds to the skeletal ring vibration of the metal porphyrin [45,46]. Furthermore, the band at around 1600 cm^−1^, which corresponds to angular deformation in the N−H plane of the pyrrole ring, should disappear, but, in this case, this cannot be observed because at this frequency, this type of vibration overlap with the band of the benzene ring vibrations. In the IR spectra of all porphyrin complexes, there is a new band at around 1660 cm^−1^; this is probably due to the carbonyl group of DMF that was used as a solvent for the dissolution of the complexes.

**Figure 5:**
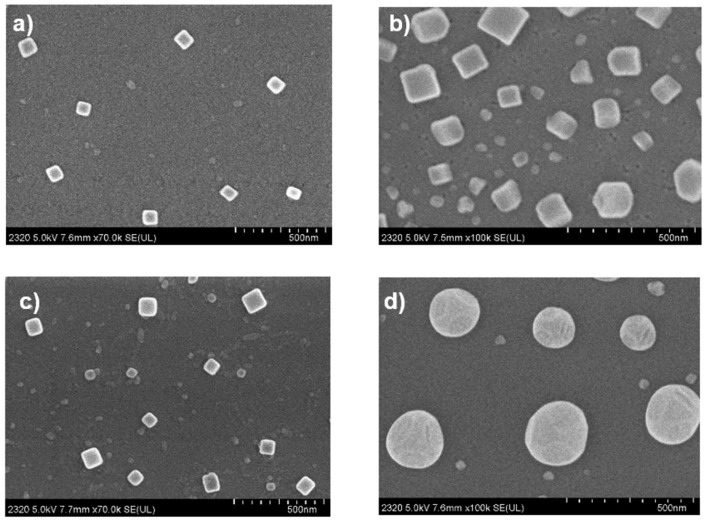
FE-SEM morphology of (**a**) Co–THPP, (**b**) Ni–THPP, (**c**) Cu–THPP and (**d**) Zn–THPP complex films deposited on glass substrate.

The morphology of cobalt, nickel, copper, zinc metalloporphyrin on the K^+^ ion-exchanged glass substrate is shown in Figure 5.

**Figure 6:**
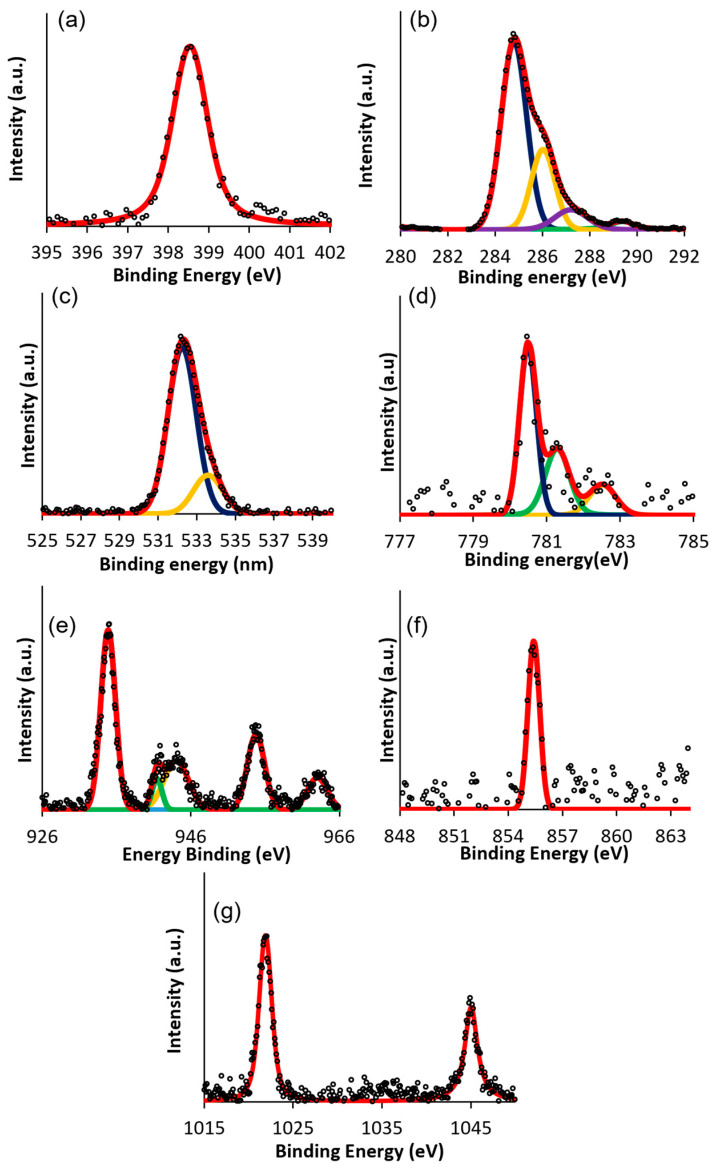
XPS spectra of porphyrin complexes (**a**) N1s, (**b**) C1s, (**c**) O1s, (**d**) Co2p_3/2_, (**e**) Cu2p, (**f**) Ni 2p_3/2_ and (**g**) Zn2p.

The metalloporphyrins, when DMF solvent vaporized, form aggregates by intermolecular force, mainly by hydrogen bond and coordination bonds on the surface of the glass [47]. All the THPP complexes show a uniform distribution but, for all the samples, are present large aggregates with distinct shapes: cubes for Co–THPP (Figure 5a), cubes and irregular shapes for Ni–THPP (Figure 5b), large cubes aggregates for the Cu–THPP (Figure 5c) while, for Zn–THPP (Figure 5d), large spheres aggregates are evident on the film. The X-ray photoelectron spectra are reported in Figure 6.

The O1s, N1sm, and C1s signals are very similar for all the M–THPP and are reported to the relative spectra in Figure 6a–c. In free-base porphyrin, N1s XPS spectrum shows two signals relative to iminic and pyrrolic nitrogens; in this case, after the metalation, as reported in Figure 6a, the nitrogen atoms become more similar, and only a single peak at around 398.5−398.6 eV is detected for all the porphyrin complexes [48]. The C1s XPS signal, reported in Figure 6b, shows different contributions at 284.8, 286, 287.2 and 289.4 eV assigned to C−C sp^2^, C = N, C−N and π−π * [49]. O1s for all the porphyrin complexes show a main peak centered at 532.80 eV relative to C−O and a smaller peak at 533.6 eV relative to -OH (Figure 6c). For Co–THPP, the Co2p_3/2_ XPS spectrum shows a main peak centered on 780.5 eV accompanied by a satellite structure at higher binding energies (Figure 6d), typical for a multilayer porphyrin film [50,51]. Cu XPS spectrum (Figure 6e) for the Cu–THPP shows two components for Cu2p_1/2_ and Cu2p_3/2_. The main peak at 935 eV is related to the 2p^3^d^10^L final state, and two satellites at 941.4 and 944 eV ascribed to a 2p^3^d^9^ final state. The Cu2p_1/2_ component shows the main peak at 955 eV and a satellite at higher binding energy 963 eV [52]. Ni–THPP XPS spectrum, reported in Figure 6f, shows a peak centered at 855.4 eV ascribed to Ni2p_3/2_ component typical for a multilayer porphyrin coverage film [53].In the case of Zn–THPP, the XPS spectrum in Figure 6g clearly shows the Zn 2p_3/2_ and Zn2p_1/2_ components at 1021.9 and 1045 eV, demonstrating the presence of the Zn–THPP porphyrin on the glass [54]. In conclusion, all the XPS spectra demonstrate the presence of the complexes in the film state, confirming that the metalation of the porphyrin ring remains stable, even after the evaporation of the solvent.

### 3.2. Interaction Analysis of Metalloporphyrin Film and NO_2_ Gas

**Figure 7:**
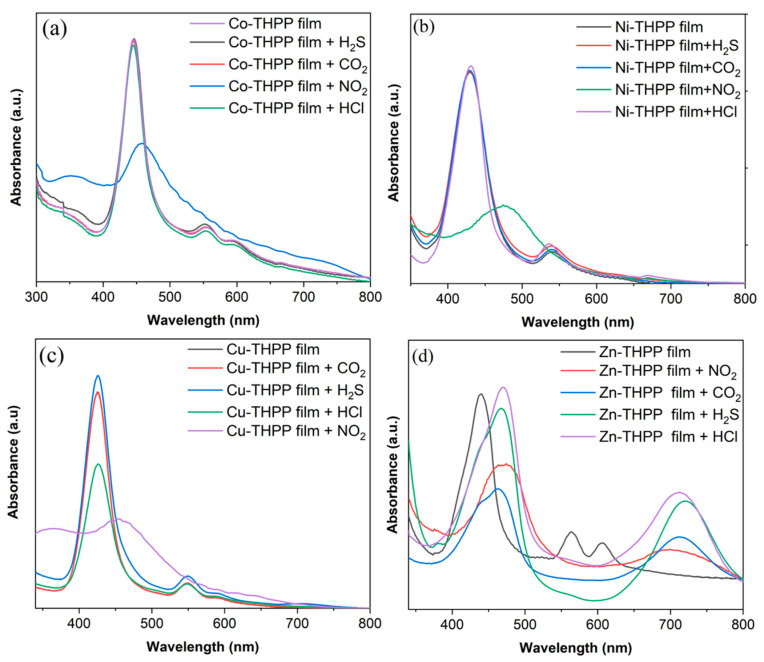
Absorption spectrum of (**a**) Co-THPP, (**b**) Ni-THPP, (**c**) Cu-THPP and (**d**) Zn-THPP complex films before and after acidic gas (1000 ppm) exposure.

The UV-Vis spectrum of each metalloporphyrin film before and after interaction with CO_2_, H_2_S, HCl, and NO_2_ (1000 ppm) is shown in Figure 7, and the obtained results are reported in Table 3. After the exposure with CO_2_, H_2_S, and HCl gases, Co–THPP, Ni–THPP, and Cu–THPP, did not show any differences in the UV-vis absorption spectra, with negligible change in shape and positions of the Soret bands and Q-bands, suggesting a very low interaction between cobalt, nickel, and copper complex films with the studied gases. However, the exposure of these porphyrin complex films with NO_2_ gas promotes changes in the shape and the position of the Soret absorption bands with an important decrease in absorbance (Figure 7a−c).

When Zn–THPP film is exposed to CO_2_, H_2_S, HCl, different spectral changes can be observed; the Soret band shifts from 440 nm to 470 nm (Δλ = 30 nm), while the two Q bands at 550−650 nm disappeared with the formation of a strong Q band at around 720−730 nm (Figure 7d). The observed spectral change, after the Zn–THPP film reacts with various acid gases, suggests that the Zn-complex is less stable under acidic conditions; in fact, the changed spectra is probably due to the characteristic protonation of porphyrin rings [43,55]. In the case of NO_2_ gas, the Soret band of Zn–THPP became broadened and shifted to a higher wavelength with a large Q-band at 700 nm, suggesting an interaction of the complex with the NO_2_ gas molecule.

**Figure 8:**
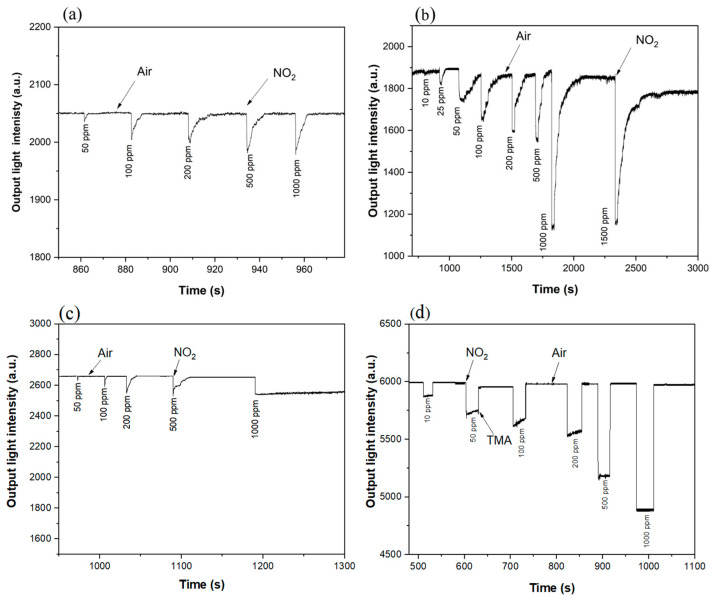
Planar optical waveguide (520 nm light source) response curve of (**a**) Co–THPP, (**b**) Ni–THPP, (**c**) Cu–THPP and (**d**) Zn–THPP porphyrin complex films exposed to NO_2_ gas with different concentration (1–1000 ppm).

Based on the interaction results of metalloporphyrin films with various acidic gases, all films were used as sensitive layers to detect NO_2_ gas with different concentrations. According to the spectral changes after interaction with NO_2_, considering the laboratory conditions, and in combining all the wavelengths with the largest changes before and after the interaction, two wavelengths of 520 nm and 670 nm were selected as excitation light sources for the optical waveguide experiment. The gas detection results of four metalloporphyrin-film-based planar optical waveguides are reported in Figure 8. 

The obtained results show that, when the wavelength of 520 nm is used as light source, the gas concentration of NO_2_ that can be detected by Co–THPP and Cu–THPP porphyrin complexes is 50 ppm, the lowest concentration can be detected by Ni–THPP and Zn–THPP porphyrin complexes and is 10 ppm.

**Figure 9:**
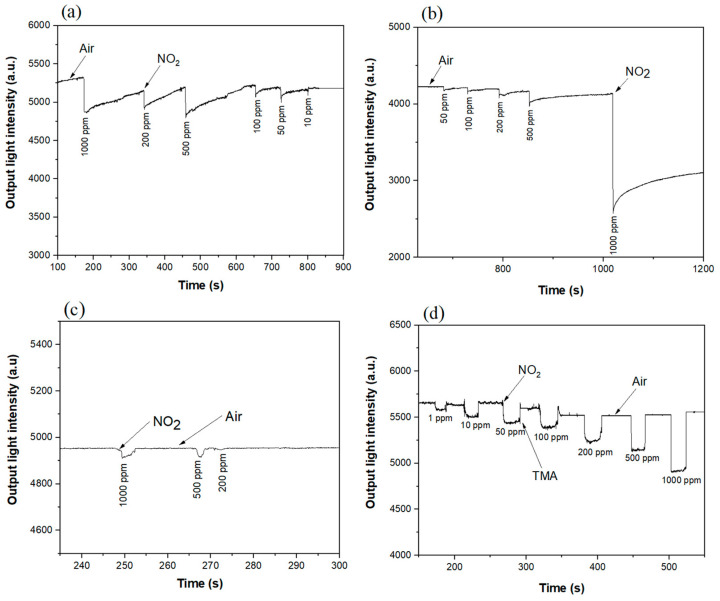
Planar optical waveguide (670 nm light source) response curve of (**a**) Co–THPP, (**b**) Ni–THPP, (**c**) Cu–THPP and (**d**) Zn–THPP porphyrin complex films exposed to NO_2_ gas with different concentration (1–1000 ppm).

However, when a 670 nm laser is used as a light source, as shown in Figure 9, 10 ppm NO_2_ gas can be detected by Co–THPP and Ni–THPP, 500 ppm by Cu–THPP, and 1 ppm by the Zn–THPP complex.

As shown in Figure 8 and Figure 9, when NO_2_-atmosphere was switched to the air atmosphere, it takes short time periods to recover naturally for Co–THPP, Ni–THPP, Cu–THPP complexes when gas concentration is lower than 1000 ppm; while in terms of the Zn–THPP complex, the NO_2_ gas exposed film is unable to recover thoroughly even after 30 min for gas concentration as low as 10 ppm, thus, in this case, it was used TMA vapor to restore the complex, confirming the reversibility of the process.

**Figure 10:**
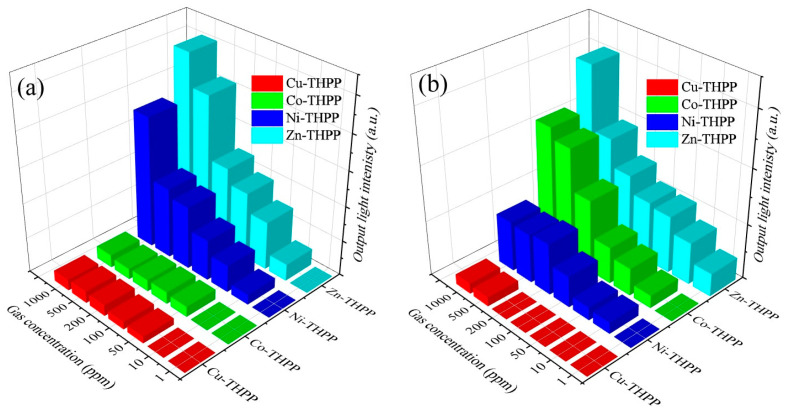
Response histogram of output light intensity on planar optical waveguide of Co, Ni, Cu, Zn porphyrin complex thin films interacting with different concentrations of NO_2_ gas (**a**) 520 nm light source (**b**) 670 nm light source.

When two wavelengths are used as light sources, and the detection results of planar optical waveguide are compared (Figure 10), it is possible to observe that low concentration (1 ppm) of NO_2_ gas can be detected only when the planar optical waveguide is fabricated with a 670 nm laser as a light source and Zn–THPP film as sensitive material.

### 3.3. Molecular Dynamic Simulations and Quantum Mechanical Calculations Analysis

For analyzing the MD data, to reveal the interaction of porphyrin molecule complexed by different metal ions with NO_2,_ the radial distribution function (RDF) was used. The radial distribution function describes the distribution of certain atoms or molecules around specific sites in particular atoms or molecules in the system. Our objective is to explore whether the change of metal ion can change the strength of interaction of NO_2_ to porphyrin molecule; therefore, we calculated the distribution of NO_2_ around different sites of porphyrin molecule in all systems.

**Figure 11:**
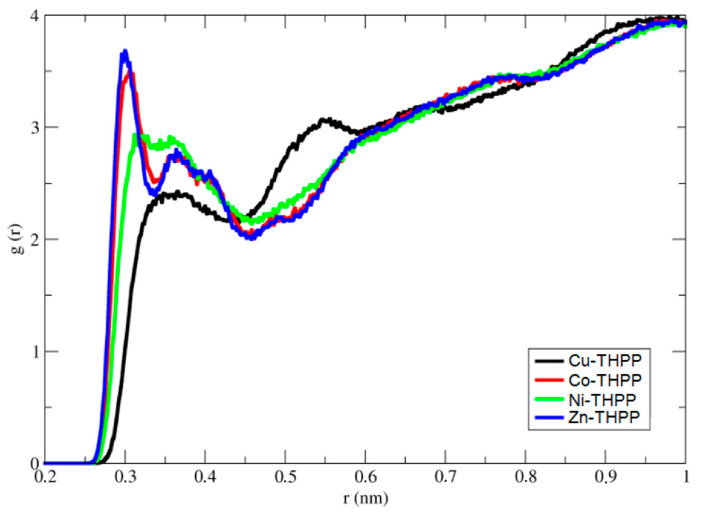
Radial distribution function of NO_2_ molecules around the center of mass of N atoms of THPP complexes with different metal ions.

Figure 11 shows the radial distribution function of NO_2_ molecules around the N atoms of porphyrin molecule in complexes with different metal ions. RDF shows that for Zn^2+^ and Co^2+^ ions, peaks with strong intensity appear at 0.3 nm, while for Ni^2+^ and Cu^2+^, much weaker peaks in intensity appear at 0.31–0.37 nm. The weaker intensities of peaks for Ni^2+^ and Cu^2+^ show a weaker interaction of NO_2_ molecules with the porphyrin molecule complexed with Ni^2+^ and Cu^2+^ metal ions. By careful investigation of the RDF, it can be concluded that the peaks with lower intensity with the broader area, which start from 0.31 nm to 0.37 nm, prove that the NO_2_ molecule can interact with a metal center in different conformations. As QM calculations showed, different energies are in accordance with different conformations. Therefore, it can be argued that in different conformers, the NO_2_ have different distances to the metal center; thus, broader peaks appear for complexes by Ni^2+^ and Cu^2+^ with porphyrin molecule.

In order to correlate the structural features of complexes of porphyrin molecules with different metal ions and the influence of conformations on the interaction energy of NO_2_ molecules in different complexes, we have calculated the interaction energy for all complexes.

**Figure 12:**
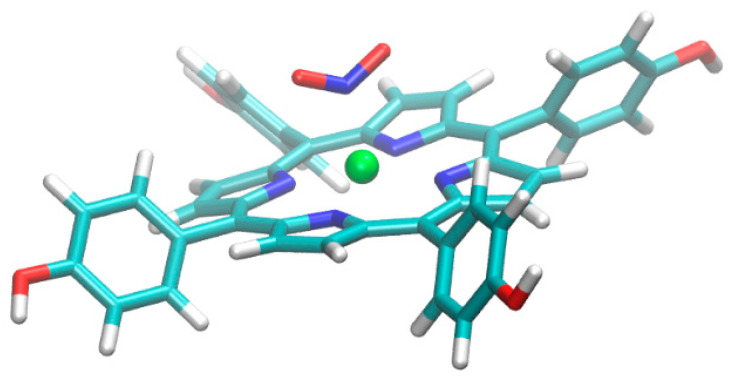
The optimized geometry of THPP porphyrin with Zn^2+^ and NO_2_ in conformation 1.

The optimized geometry of THPP with Zn^2+^ and NO_2_ in conformation one, where N atom interacts with metal, is presented in Figure 12.

**Figure 13:**
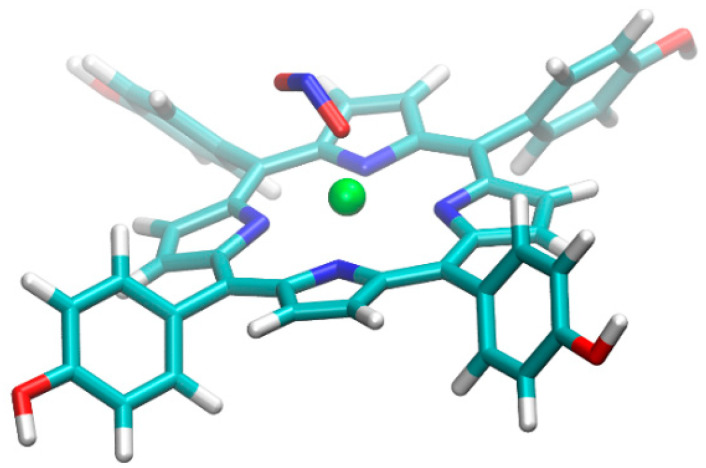
The optimized geometry of THPP porphyrin with Zn^2+^ and NO_2_ in conformation 2.

In conformation one, the geometry of the complexes with the other metal ions Ni^2+^, Co^2+^, and Cu^2+^ has not been significantly changed after their geometrical re-optimization. The optimized geometry of THPP porphyrin with Zn^2+^ metal ion and NO_2_ in conformation two, where oxygen atom interacts with Zn^2+^ metal ions, is presented in Figure 13. The gas-phase interaction energies of the complexes between THPP porphyrin and NO_2_ molecule calculated for different conformations are presented in Table 4.

From the results, it can be concluded that for the complexes at the same geometrical conformation, the Cu–THPP does not interact with NO_2_ in conformation one, while the strongest interaction was observed for Ni–THPP with NO_2_. However, in conformation two, where O atom interacts with metal ions, QM calculations revealed that all complexes have lower interaction energies, even the Cu–THPP, which shows very low interaction with NO_2_; with this conformation, the strongest interaction occurs for Co–THPP and Zn–THPP.

## 4. Conclusions

Four complexes of THPP porphyrin (Co, Cu, Ni, and Zn) were deposited as film on K^+^-exchanged glass and used in planar optical waveguides for the investigation on the detection of several gases such as HCl, H_2_S, CO_2_, and NO_2_ at 20 °C. UV−Vis absorption spectra of these thin films exposed to analyte gases showed that all these films display remarkable absorption change with NO_2_, whereas not observable difference with other gases except for the Zn–THPP complex, which exhibits obvious changes with HCl, H_2_S, CO_2_ gas exposures as well. Since Cu–THPP, Co–THPP, and Ni–THPP complexes are not sensitive to other gas, except for NO_2_, in this work, we focused on the response behavior of the four complexes towards NO_2_ gas. With the fabricated devices, the amount of the lowest detectable NO_2_ gas differs as 50 ppm with Co–THPP and Cu–THPP complexes, while 10 ppm with the Ni–THPP complex and the Zn–THPP complex under 520 nm laser light illumination. When the laser illumination was at 670 nm, the device with the Zn–THPP complex was able to detect 1 ppm, while Co–THPP-, Ni–THPP- and Cu–THPP-based devices were able to detect 10 ppm, 50 ppm, and 500 ppm of NO_2_ gas, respectively. By combining the results of the molecular dynamics simulation and quantum mechanical calculation, it is possible to observe that the Cu–THPP complex shows the least affinity toward NO_2_ gas molecules, demonstrated by the binding energies that are close to zero, suggesting there is a very low or no interaction between copper complex and NO_2_. Compared with other M–THPP metalloporphyrins, the affinity of Cu–THPP to NO_2_ is very low by the optical waveguide detection, in agreement with MD and quantum mechanical calculation.

Nickel, cobalt, and zinc complexes display good sensitivity in the UV-Vis spectrum leading to sensible absorbance changes at 520 nm or 670 nm wavelength after NO_2_ exposure. This is inconsistent with the binding energy calculation of these three complexes interacting with NO_2_ molecules, that is, Ni–THPP −14.9 kcal/mol, Co–THPP −11.8 kcal/mol, Zn–THPP −11.0 kcal/mol in configuration one, and Ni–THPP −19.9 kcal/mol, Co–THPP −30.6 kcal/mol and Zn–THPP −29.7 kcal/mol in terms of the configuration 2. In optical waveguide detection, Co–THPP and Ni–THPP show a lower response than Zn–THPP; this could be ascribed to the higher absorbance changes for Zn–THPP at 520 and 670 nm after NO_2_ interaction. All experimental results show that the better wavelength to detect the interaction between M–THPP and NO_2_ gas is 670 nm, which demonstrating the lowest detectable amount of NO_2_ gas for all the THPP complexes.

## Figures and Tables

**Table 1:** Absorption spectrum data and calculated molar extinction coefficient results of porphyrin complexes in DMF solution.

	Soret Band		Q Band			
Complexes	λ (nm)	ε (M^−1^ cm^−1^)	Λ (nm)	ε (M^−1^ cm^−1^)	Λ (nm)	ε (M^−1^ cm^−1^)
Co–THPP	420	2.87 × 10^5^	534	1.09 × 10^4^	590	3.19 × 10^3^
Ni–THPP	421	1.38 × 10^5^	531	9.15 × 10^4^	570	2.01 × 10^3^
Cu–THPP	421	9.38 × 10^5^	542	4.42 × 10^4^	582	1.30 × 10^4^
Zn–THPP	429	5.00 × 10^8^	562	1.46 × 10^7^	604	1.14 × 10^7^

**Table 2:** UV-vis spectrum properties of porphyrin complexes in solution (DMF) and film state.

Complexes	λ_Soret_ Solution (nm)	λ_Soret_ Film (nm)	Shift Δλ (nm)
Co–THPP	420	446	26
Ni–THPP	421	431	10
Cu–THPP	421	429	8
Zn–THPP	429	440	11

**Table 3:** Absorption analysis of THPP complex films with NO_2_ gas (1000 ppm) exposure.

	Soret Band		Q Band	
Complexes	Before	After NO_2_	Before	After NO_2_
Co–THPP	445	455	553, 596	-
Cu–THPP	427	448	550	-
Ni–THPP	431	474	536	-
Zn–THPP	440	474	563, 607	700

**Table 4:** The Interaction energies between THPP complexes and NO_2_ calculated at B3LYP/6-31G* level.

System	Interaction Energy [kcal/mole]	Interaction Energy [kcal/mole]
	Conformation 1	Conformation 2
NO_2_—Zn–THPP	−11.0	−29.7
NO_2_—Cu–THPP	+0.33	−2.98
NO_2_—Co–THPP	−11.8	−30.6
NO_2_—Ni–THPP	−14.9	−19.9

## Data Availability

The data that support the findings of this study are available from the corresponding author, upon reasonable demand.
